# Socio-demographic factors associated with smoking and smoking cessation among 426,344 pregnant women in New South Wales, Australia

**DOI:** 10.1186/1471-2458-5-138

**Published:** 2005-12-21

**Authors:** Mohammed Mohsin, Adrian E Bauman

**Affiliations:** 1 Biostatistician, Centre for Research, Evidence Management & Surveillance, Division of Population Health, Sydney South West Area Health Service, Liverpool BC, NSW 1871, Australia; 2 Professor of Public Health & Epidemiology, School of Public Health, University of Sydney & University of New South Wales, Australia

## Abstract

**Background:**

This study explores the socio-demographic characteristics of pregnant women who continue to smoke during the pregnancy, and identifies the characteristics of the smokers who were likely to quit smoking during the pregnancy period.

**Methods:**

This was secondary analysis of the New South Wales (NSW) Midwives Data Collection (MDC) 1999–2003, a surveillance system covering all births in NSW public and private hospitals, as well as home births. Bivariate and multiple logistic regression analyses were performed to explore the associations between socio-demographic characteristics and smoking behaviour during pregnancy.

**Results:**

Data from 426,344 pregnant women in NSW showed that 17.0% continued to smoke during pregnancy. The smoking rate was higher among teenage mothers, those with an Aboriginal (indigenous) background, and lower among more affluent and overseas-born mothers. This study also found that unbooked confinements, and lack of antenatal care in the first trimester were strongly associated with increased risk of smoking during pregnancy. About 4.0% of the smoking women reported they may quit smoking during their pregnancy. Findings showed that mothers born overseas, of higher socio-economic status, first time mothers and those who attended antenatal care early showed an increased likelihood of smoking cessation during pregnancy. Those who were heavy smokers were less likely to quit during pregnancy.

**Conclusion:**

Although the prevalence of smoking during pregnancy has been declining, it remains a significant public health concern. Smoking cessation programs should target the population subgroups of women at highest risk of smoking and who are least likely to quit. Effective antismoking interventions could reduce the obstetric and perinatal complications of smoking in pregnancy.

## Background

The harmful effects of smoking during pregnancy was first investigated by Spontag and Wallace in 1935 [1]. They reported 'a definite and real' increase in fetal heart rate after the mother began to smoke. Since then there is clear evidence of risks associated with smoking in pregnancy from epidemiological, clinical and experimental studies [2-4].

Smoking in women has been shown to be detrimental to fertility [5], and to increase the maternal and fetal risks during pregnancy and the perinatal period [6]. Recent epidemiological reviews have suggested that smoking in pregnancy (SIP) increases the incidence of maternal complications such as placental abruption, placenta previa, ectopic pregnancy, prolonged rupture of membranes, inflammation of the umbilical cord, and amniotic fluid bacterial infections [4,7,8]. There is a well-established association between maternal smoking and reduced birth weight, which may lead to subsequent growth delay [4,9-11]. SIP is also associated with increased rates of postnatal respiratory infection, wheeze and otitis media [12,13], as well as being an identified risk factor for sudden infant death syndrome [14-16]. Studies on the economic consequences of smoking during pregnancy suggest that smoking-attributable costs related to low birth weight is enormous, largely due to higher admission rates to neonatal intensive care units [17,18]. Studies also reported 66% higher medical costs for complicated births for smoking mothers compared to non-smoking mothers[19].

Maternal smoking during pregnancy may also have intergenerational effects. Kandel et al. [20] followed a sample of mothers who smoked during pregnancy for over 19 years, and showed higher rates of smoking among their female offspring, after adjustment for suspected confounders. It was suggested that maternal smoking may have had a biological effect, predisposing the fetus to nicotine dependence, and increasing the risk of daughters smoking [21].

Although recent studies suggest that the prevalence of SIP has been declining, it remains high in many countries, with between one in seven and one in three pregnant women in developed countries continuing to smoke [22,23,10]. In Canada, SIP rates were 30% in Saskatoon and 32% in Nova Scotia in early 1990s, with the highest rates among low socio-economic status (SES) and younger mothers, and it declined to 17.8% (Southern Ontario) in 2001 [24-26]. British data suggest SIP rates declined from 37% in 1989 to 22.4% in 2005 [18,27]. US data suggest an overall rate slightly lower than this, with rates declining from 18.4% in 1990 to 11.4% in 2002 [17,28].

Long term data from Finland estimated 22% smoking in 1966, which declined to 18% in 1987 and ten years later it remained similar, with 15% of mothers smoking in 1997 [28,29]. Some evidence of a recent decline in prevalence from 34% in the mid 1980s to 22% in 1994 was reported in Norway [30].

SIP has been shown to be associated with education level, maternal age, social class and lack of private health insurance [27,31,32]. Typically a third of smokers report that they cut down or quit smoking during their pregnancy [33,34], although the number who self-report that they smoke during pregnancy may be an underestimate of the true prevalence [35].

Smoking in pregnancy remains prevalent in Australia [9,10], and further investigation of the correlates and population groups at risk are required to develop population-level targeted interventions. This paper reports data on prevalence and on the factors associated with smoking in pregnancy among all births in NSW between 1999 and 2003. This provides a large, comprehensive sample of women who delivered babies in the state, and was collected using a population-based surveillance system. The aims of this investigation were (i) to identify socio-demographic factors associated with smoking in pregnancy in NSW, (ii) to compare those who quit (smoking cessation) during pregnancy with those who continue, [iii] to compare the characteristics of heavy versus light smokers in late pregnancy, and [iv] to compare these results with smoking prevalence rates in general population surveys of all women of childbearing age. The study aims to identify the highest risk population segments, in order to develop group-specific interventions.

## Methods

The data were the NSW Midwives Data Collection (MDC) 1999–2003, a surveillance system covering all births in NSW public and private hospitals, as well as home births. The MDC is administered by the NSW Health Department, and encompasses all live-births and stillbirths of at least 20 weeks gestation or at least 400 grams birth-weight. It relies on the attending midwife to complete a notification form when a birth occurs. The form includes demographic items and items on maternal health, smoking, the pregnancy, labour, delivery and perinatal outcomes. The validation study on the NSW MDC system compared records of raw MDC as reported by hospitals with the hospital medical record [36]. Excellent levels of agreement were demonstrated for all the 44 data items studied. The majority of the data items had a 90% or more agreement.

Pregnant women were classified as smokers or non-smokers according to their self-reported smoking status during pregnancy. They were classified as 'smokers' if they ever smoked during the current pregnancy. Categories of tobacco use were based upon the reported quantity smoked in the second half of pregnancy: (1) quitters, who reported smoking at the commencement of pregnancy, but did not smoke in the second half of pregnancy; (2) light smokers, reported 10 cigarettes or fewer per day; (3) heavy smokers, reported more than 10 cigarettes per day; and (4) smoking quantity unknown. Patients socio-economic status (SES) was constructed based on 'the Index of socio-economic disadvantage' calculated for NSW 2001 census population by postcode of residence. Firstly, all the postcodes for NSW MDC were ranked by index of socio-economic disadvantage, and then grouped into five quintiles of SES from the lowest to the highest. Finally, these were grouped in to three SES categories: lowest SES (lowest and the second lowest quintiles of SES), moderate SES (3^rd ^and 4^th ^quintiles of SES) and Highest SES (the 5^th ^quintile of SES).

Comparisons were carried out between these NSW MDC data and a representative population survey. This was the NSW Continuous Health Survey program 2002–2003, which asked a random sample of 25,630 NSW adults about a range of behaviours including smoking (NSW Continuous Health Survey 2002 onwards NSW Health 2004). Raw data from the NSW study was reanalysed, with females chosen only, and age groups 18 up to 20 years, 20–34, and 35–44 years to assess smoking prevalence. Weighted prevalence estimates were reported.

Bivariate analyses were used to explore the associations between socio-demographic characteristics and smoking during pregnancy. Chi-square tests were used to test for statistical significance. Multiple logistic regression analyses were constructed to examine the relative contribution of each of the socio-demographic variables on smoking behaviour during pregnancy. Based on the initial analyses, factors placing groups at highest risk of smoking in pregnancy were further examined, to identify sub-groups at extreme risk in the population. Separate logistic regression models were constructed to examine factors associated with attempts at smoking cessation during pregnancy, and light versus heavy smokers. Adjusted odds ratios (OR) and 95% confidence intervals (CI) were used, and the log likelihood and model chi square were used to examine the goodness-of-fit and adequacy of the logistic regression models.

## Results

### Socio-demographic characteristics of smoking in pregnancy

Of the 426,425 women confined in NSW 1999–2003, there were 426,344 women whose smoking status was recorded. Among them 72,428 were smokers (17.0%), and 353,916 were non-smokers. The demographic characteristics of these women are presented by age, ethnicity, parity, socio-economic status and hospital booking for confinement (Table 1).

**Table 1:** Socio-demographic determinants of smoking during pregnancy: percentage of smoking and adjusted odds ratios (AOR) from multiple logistic regression analysis

Socio-demographic characteristics	Total number of women (%)	% of smokers (n = 72,428)	^Ψ^Adjusted odds ratio (95% CI)
Year of delivery#			
1999–2003	426344 (100.0)	17.0	
1999	85655 (20.2)	19.0	1.00
2000	86439 (20.3)	17.4	0.91 (0.88–0.93)*
2001	84362 (19.8)	17.1	0.87 (0.85–0.89)*
2002	84573 (19.8)	16.4	0.83 (0.81–0.85)*
2003	85015 (19.9)	15.1	0.77 (0.75–0.79)*
Maternal age (years)#			
Under 20 years	18778 (4.4)	42.9	3.73 (3.58–3.89)*
20–34	329834(77.4)	16.9	1.31 (1.27–1.34)*
35+	77553(18.2)	11.2	1.00
Aboriginal status#			
Non Aboriginal	415583(97.5)	16.0	1.00
Aboriginal	10579 (2.5)	57.8	3.43 (3.29–3.58)*
Parity#			
Primiparous	177304 (41.6)	14.3	1.00
Multiparous	248991 (58.4)	18.9	1.49 (1.46–1.51)*
Country of birth#			
Australian born	309580 (72.7)	20.5	1.00
NZ and Oceania	17809(4.2)	18.9	0.75 (0.72–0.78)*
UK & Ireland	12866 (3.0)	11.6	0.77 (0.73–0.82)*
Other Europe	11195 (2.6)	10.1	0.51 (0.47–0.54)*
Middle East	16864 (4.0)	8.3	0.25 (0.24–0.27)*
Asia	46721 (11.0)	1.8	0.066 (0.06–0.07)*
America/Africa	7313 (1.7)	5.2	0.24 (0.22–0.26)*
Others	3680 (0.9)	7.0	0.44 (0.39–0.50)*
Socio-economic status (SES)#			
Lowest SES	166987 (39.5)	22.3	3.33 (3.23–3.43)*
Moderate SES	168661 (39.9)	16.9	2.40 (2.32–2.47)*
Highest SES	87404 (20.7)	6.6	1.00
Booking in#			
Booked	416223 (97.6)	16.5	1.00
Unbooked	10120(2.4)	36.7	1.52 (1.44–1.61)*
Weeks at 1st antenatal visit#			
0–12 weeks	245729 (58.2)	14.4	1.00
13–26 weeks	156396 (37.0)	18.6	1.45 (1.42–1.47)*
27 weeks +	20314(4.8)	31.8	2.68 (2.58–2.77)*

#Statistically significant (p < 0.05) in bivariate analyses; * ORs significant at p < 0.05 level; ^Ψ^The fitted multiple logistic regression model is significant at p < 0.0001 (Model Chi square = 44067.74, df = 20), The model predicted overall 83.7% correctly classified (considering cut point 0.50). Dependent variable: Current smoking status (smoked yes = 1, no smoking = 0), Explanatory variables :Year of delivery, maternal age, Aboriginal status, parity, country of birth, SES, booking in and weeks of gestation at first antenatal care. For all variables not stated and missing cases excluded from the analyses.

Only 4.4% of confinements were to mothers aged less than 20 years, and 18.2% were to those aged 35 years or older. The majority of the women were Australian born (72.7%), followed by other ethnic groups. Of the women, 41.6% gave birth for the first time, 1.5% were unbooked for this confinement and 2.5% were of indigenous (Aboriginal) background (Table 1). Overall, more than half (58.2%) of mothers attended their first antenatal visit in the first trimester of pregnancy, 37.0% in the second trimester and 4.8% attended in the third trimester.

The relationship between various maternal characteristics and smoking during pregnancy is shown in table 1. The sample size for each subgroup is shown in the left hand column, and the smoking prevalence in the middle column. The adjusted odds ratios with 95% confidence interval (95% CI) for smoking, compared to the reference group (OR = 1.00) are shown in the right hand column. In 1999–2003, 17% of the NSW mothers smoked during the pregnancy period and this rate declined from 19% in 1999 to 15.1% in 2003. The results showed much higher rates of smoking during pregnancy among teenage mothers (42.9% smoked), compared to older mothers over 35 years, of whom 11.2% smoked. Even among young teenage mothers aged 16 years or less, almost half smoked during pregnancy. High rates of smoking were reported by Aboriginal mothers (57.8%), who were over three times as likely to smoke compared to non-Aboriginal mothers (OR = 3.43, 95%CI: 3.29–3.58). On the other hand, overseas-born mothers showed low to very low rates of smoking. Compared with the 20.5% rate among Australia-born women, all immigrant women were less likely to smoke, with only 1.8% among Asian born women (Odds Ratio = 0.06).

Multiparity, lower socio-economic status, unbooked confinements, and lack of antenatal care in the first trimester were other factors significantly and independently associated with increased risk of maternal smoking during pregnancy.

### Identifying sub-groups at extreme risk

Based on the analyses in table 1, the three groups at highest risk of smoking in pregnancy were younger mothers, indigenous, and late antenatal care attenders. These three groups are examined in table 2, exploring further the interactions between them, in order to identify the highest risk sub-groups. Although there was a strong dose response gradient in SIP rates by age among non-Aboriginal mothers, this age-relationship was much less apparent among Aboriginal mothers, where SIP rates ranged from 56.7% to 60.5% by age. For early (<12 weeks) antenatal care attenders, there was a strong relationship with age (a fourfold decrease in SIP rates across age groups), but for those attending antenatal care latest, there was much less of a gradient. There was a three to fourfold gradient in SIP rates between Aboriginal and non-Aboriginal mothers, irrespective of antenatal care attendance. By socio-economic status, the gradient in SIP prevalence was greatest for older mothers, and least for teenage mothers, and the gradient was greater among non-Aboriginal mothers compared to Aboriginal mothers. There were relatively fixed differentials between English speaking and non-English speaking background mothers, irrespective of age or antenatal care.

**Table 2:** Defining the interactions among high risk groups for smoking in pregnancy in NSW

	Percentage of smoking during pregnancy (SIP)							
	
	Maternal age (years)			Aboriginal status		Weeks of gestation at 1st antenatal visit		
	
	<20 years	20–34	35+	Aboriginal	Non-Aboriginal	0–12	13–26	27+
Maternal age (years)								
<20 years	-	-	-	60.5	40.5	43.0	41.6	44.7
20–34	-	-	-	56.7	15.9	14.3	18.3	31.4
35+	-	-	-	60.2	10.7	9.4	12.7	24.6
Aboriginal status								
Aboriginal	60.5	56.7	60.2	-	-	51.3	58.2	72.5
Non-Aboriginal	40.5	15.9	10.7	-	-	13.6	17.6	28.7
Socio-economic status (SES)								
Lowest SES	42.8	21.5	17.4	59.8	20.7	20.1	22.1	34.4
Moderate SES	43.7	16.6	11.5	55.1	16.0	14.5	18.8	32.1
Highest SES	36.3	6.9	4.8	36.5	6.5	5.0	9.1	16.3
Country of birth								
English speaking background	45.7	19.8	13.5	-	-	16.1	23.9	45.0
Non-English speaking background	10.3	4.9	4.8	-	-	3.8	5.2	9.4
Weeks of gestation at 1st antenatal visit								
0–12 weeks	43.0	14.3	9.4	51.3	13.6	-	-	-
13–26 weeks	41.6	18.3	12.7	58.2	17.6	-	-	-
27 weeks +	44.7	31.4	24.6	72.5	28.7	-	-	-

### Smoking cessation (quit) and amount smoked during pregnancy

Among the 72,428 pregnant women who identified themselves as smokers, 2,920(4.0%) reported quitting during pregnancy and this rate declined from 4.5% in 1999 to 3.3% in 2003. The prevalence rates and odds ratios for various maternal characteristics associated with smoking cessation during pregnancy are shown in table 3. The following factors were significantly associated with an increased likelihood of smoking cessation during pregnancy: mothers who were born in Asia, first time mothers, higher SES group, those who reported early attendance for antenatal care and those with any obstetric complication.

**Table 3:** Socio-demographic determinants of smoking cessation and heavy smoking during pregnancy: percentage of quit, % of heavy smokers and adjusted odds ratios (AOR) from multiple logistic regression analysis

Socio-demographic characteristics	Total number of smokers	% of smokers quit smoking (n = 2,920)	^Ψ^Adjusted odds ratio (95% CI)	% of heavy smokers	^Ψ^Adjusted odds ratio (95% CI)
Year of delivery#					
1999–2003	69500	4.0		49.6	
1999	16302	4.5	1.00	52.2	1.00
2000	15000	4.1	0.92 (0.83–1.04)	49.7	0.90 (0.86–0.94)*
2001	14422	4.0	0.92 (0.82–1.03)	49.6	0.89 (0.85–0.93)*
2002	13820	4.0	0.94 (0.83–1.05)	48.9	0.85 (0.81–0.89)*
2003	12875	3.3	0.77 (0.68–0.87)*	46.8	0.78 (0.76–0.82)*
Maternal age (years)#					
Under 20 years	8063	3.9	0.84 (0.71–0.99)*	45.4	1.00
20–34	55625	4.1	1.11 (0.98–1.26)	49.6	1.01 (0.95–1.06)
35+	8705	3.6	1.00	53.0	1.18 (1.10–1.27)*
Aboriginal status#					
Non Aboriginal	66287	4.3	1.86 (1.51–2.30)*	48.7	1.00
Aboriginal	6112	1.6	1.00	58.6	1.24 (1.17–1.31)*
Parity#					
Primiparous	25407	6.8	2.55 (2.34–2.77)*	40.2	1.00
Multiparous	47007	2.6	1.00	54.4	1.68 (1.62–1.73)*
Country of birth#					
Australian born	63519	4.0	1.00	50.7	1.00
NZ and Oceania	3374	3.4	0.94 (0.77–1.14)	41.3	0.61 (0.57–0.66)*
UK & Ireland	1488	4.7	0.90 (0.70–1.16)	47.6	0.94 (0.84–1.04)
Other Europe	1129	4.1	0.83 (0.61–1.12)	40.4	0.68 (0.60–0.78)*
Middle East	1406	2.8	0.87 (0.63–1.21)	41.3	0.56 (0.51–0.64)*
Asia	822	10.9	2.71 (2.15–3.43)*	28.1	0.37 (0.31 – 0.44)*
America/Africa	380	7.4	1.38 (0.91–2.1)	35	0.56 (0.44–0.69)*
Others	258	5.6	1.00(0.57–1.76)	45.9	0.89 (0.69–1.17)
SES#					
Lowest SES	37289	3.1	1.00	52.7	1.84 (1.72–1.96)*
Moderate SES	28485	3.8	1.11 (1.02–1.21)*	48.0	1.58 (1.48–1.68)*
Highest SES	5797	10.2	2.79 (2.51–3.10)*	35.8	1.00
Booking in#: Booked	68710	4.2	1.83 (1.33–2.53)*	48.9	1.00
Unbooked	3710	1.3	1.00	62.0	1.20 (1.10–1.31)*
Weeks at 1^st ^antenatal visit#					
0–12 weeks	35263	5.0	2.33 (1.91–2.85)*	46.7	1.00
13–26 weeks	29084	3.6	1.75 (1.42–2.14)*	50.1	1.17 (1.13–1.21)*
27 weeks +	6453	1.7	1.00	58.3	1.55 (1.47–1.66)*
Any obstetric condition#: No	67272	3.9	1.00	48.9	1.00
Yes	5148	5.4	1.19 (1.04–1.35)*	62.0	1.20 (1.10–1.31)*

#Statistically significant (p < 0.05) in bivariate analyses; * ORs significant at p < 0.05 level; The fitted multiple logistic regression model is significant at p < 0.001; The model predicted overall 95.9% correctly classified (considering cut point 0.50). For all variables not stated and missing cases excluded from the analyses.

Although no questions were asked about tobacco reduction, all smokers were asked about the amount they smoked during pregnancy. Smokers were classified as heavy smokers (>10 cigarettes per day), or light smokers (smoked less than 10 daily) and % of heavy smokers by socio-demographic characteristics are shown in Table 3. Of the total smokers 34,318 (50.4%) reported to be light smokers and 33720 (49.6%) were heavy smokers and those whose quantity of smoking were unknown are excluded. Although smoking rates among older mothers (35+ years) were lower, they were more likely to be heavy smokers than teenage mothers (53% vs. 45.4%). This difference was significant in univariate analysis, and 18% more likely in the adjusted analysis (p < 0.05). Heavy smoking was more likely among Aboriginal mothers, in those with lower SES, multiparous, unbooked mothers and late antenatal care attenders. The highest proportion of heavy smokers was among unbooked mothers who smoked (62%) and the lowest were among the Asian women (28.1%).

### Comparisons with general population surveys

**Figure 1:**
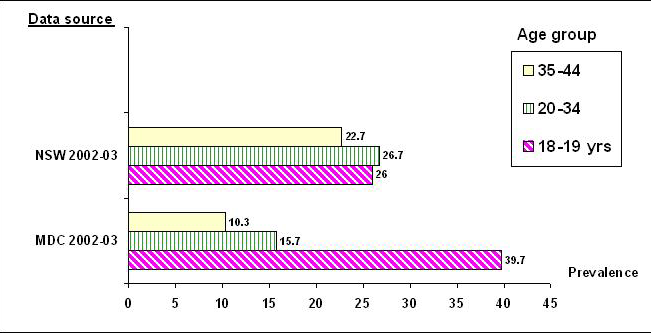
Comparisons of smoking prevalence among women of childbearing age: smoking during pregnancy from the NSW MDC (2002-2003) compared to women from population samples from NSW continuous health surveys (2002-2003)

The analysis between MDC women 2002–2003 and age matched women from general population surveys (NSW continuous health surveys 2002–2003) are shown in figure 1. Teenage smoking in pregnancy data from the MDC were confined to those aged 18–19 years for age group comparability with the general population surveys. Teenage smoking in pregnancy was much higher (39.7%) than general sample of age matched NSW data (26%). For mothers aged 20–34 or 35 and older, smoking rates in pregnancy were typically about a third lower than smoking rates among age matched women in the general community, suggesting that about a third might have quit before or early in pregnancy.

## Discussion

This study explored the socio-demographic characteristics of women who continue to smoke during pregnancy. In 1999–2003, 17% of all NSW pregnant women smoked during their pregnancy period and it declined from 19% in 1999 to 15.1% in 2003. Previous studies on NSW reported more than one-fifth of the pregnant women smoked during pregnancy period [4,10]. Although the prevalence of smoking during pregnancy has been declining, it remains a significant public health concern. The findings showed that maternal social and demographic characteristics were significantly associated with smoking behaviour. The smoking rate was worryingly high among teenage mothers, a finding consistent with previous studies in NSW and overseas [10,27]. The highest-risk group analyses suggest that strongest influencers for SIP might include maternal age and indigenous status, with half of some of these subgroups reporting smoking in pregnancy, a rate four times the general population level.

On the other hand, overseas-born mothers showed low to very low rates of smoking, and based on the data in table 2, a protective effect independent of maternal age. Multiparity, lower SES, unbooked confinements, and lack of antenatal care in the first trimester were other factors significantly and independently associated with increased risk of maternal smoking during pregnancy. Similar to the Canadian study, pregnant smokers in this study were more likely to begin prenatal care in the second or third trimester, or to receive no care at all [24-26]. These socio-economic markers identify groups at risk of sub optimal antenatal care, of which smoking is one important indicator [37].

About 4% of women reported that they may quit smoking during pregnancy. Mothers who were born in Asia, first time mothers, those who were of higher SES and those who reported early attendance for antenatal care reported an increased likelihood of smoking cessation during pregnancy: Heavy smoking (more than 10 cigarettes) was common among older mothers (aged over 35). The later group may reflect greater duration of nicotine exposure and greater dependence. The results also showed that Aboriginal mothers, lower SES, multiparous, unbooked mothers and late antenatal care attenders were more likely to be heavy smokers. The results showed that over the years, the rate of smoking during pregnancy and proportion of heavy smokers declined. From these findings one might expect the rate of intention to quit to increase, but the reverse was observed, as the rate of quitting also declined.

The analysis between MDC women and age matched women from general population surveys showed that teenage smoking in pregnancy was much higher than general sample age matched NSW data. This suggests substantial differences, in that teenagers who become pregnant are risk takers (and also at increased risk for alcohol and other drug usage) [38]. It suggests that smoking in pregnancy, among teenage mothers, may be a marker of broader health compromising risk taking, which may have adverse maternal and infant effects. For mothers aged 20–34 or 35 and older, smoking rates in pregnancy were typically about a third lower than smoking rates among age matched women in the general community, suggesting that about a third might have quit before or early in pregnancy.

About 20% to 40% of women quit smoking on their own initiative when they realise they are pregnant [39]. However, there remains a large proportion of pregnant smokers who are unable to quit. It is clear that smoking cessation is a complex undertaking which requires specific skills [40].

Kendrick et al. [41] randomly assigned clinics to intervention or control status across three states, and observed higher self-reported quitting among intervention clinics. However, the cotinine verified quit rates were not significantly different. This study highlighted that pregnant smokers may react to smoking cessation counselling by giving the desired response to questions at follow-up, and the difficulties in integrating such a program in public prenatal clinics where existing staff were already busy. Another study [42] reported that adding individual smoking cessation counselling did not increase quitting rates during pregnancy. Apart from the materials used in various interventions, partners of the pregnant smokers may have played a role in successful smoking cessation. Women who have a non-smoking partner, or living in a non-smoking household, or encouraged by their partners to stop smoking, were found to have a strong association with successful cessation [43,44]. There are challenges in contributing to further effective smoking cessation interventions, which may need to be specifically targeted at the sub-groups identified here.

A previous study in Australia estimated that if smoking were eliminated, 19.8% of the total low birth-weight incidence, 7.8% of the preterm births and 3.6% of admission require to special care nursery or neonatal intensive care unit (SCN/NICU) would have been prevented [4]. Researchers have re-iterated identified the poor obstetric outcomes for women who smoked during the pregnancy [4,9-11,45,46], making investment in exploring SIP interventions a public health priority. However, the remaining smokers may be difficult to change, and interventions may require larger effect sizes than have been produced to date [47].

## Conclusion

The social environment, and individual level demographic attributes are shown to be significantly associated with smoking behaviour and quitting smoking during pregnancy. More innovative system wide approaches to smoking in pregnancy are required, especially among teenage mothers, those at social disadvantage or those from an Aboriginal background. The information provided in this paper challenges policy makers to develop innovative interventions for those at highest risk.

## Competing interests

The author(s) declare that they have no competing interests.

## Authors' contributions

MM was responsible for data analysis and led the writing of the manuscript. MM and AEB drafted and revised the manuscript.

## Pre-publication history

The pre-publication history for this paper can be accessed here:


